# End-sequencing and characterization of silkworm (*Bombyx mori*) bacterial artificial chromosome libraries

**DOI:** 10.1186/1471-2164-8-314

**Published:** 2007-09-07

**Authors:** Yoshitaka Suetsugu, Hiroshi Minami, Michihiko Shimomura, Shun-ichi Sasanuma, Junko Narukawa, Kazuei Mita, Kimiko Yamamoto

**Affiliations:** 1 National Institute of Agrobiological Sciences, 1-2 Owashi, Tsukuba, Ibaraki 305-8634, Japan; 2 Mitsubishi Space Software Co. Ltd., 1-6-1 Takezono, Tsukuba, Ibaraki 305-0032, Japan

## Abstract

**Background:**

We performed large-scale bacterial artificial chromosome (BAC) end-sequencing of two BAC libraries (an *Eco*RI- and a *Bam*HI-digested library) and conducted an *in silico *analysis to characterize the obtained sequence data, to make them a useful resource for genomic research on the silkworm (*Bombyx mori*).

**Results:**

More than 94000 BAC end sequences (BESs), comprising more than 55 Mbp and covering about 10.4% of the silkworm genome, were sequenced. Repeat-sequence analysis with known repeat sequences indicated that the long interspersed nuclear elements (LINEs) were abundant in *Bam*HI BESs, whereas DNA-type elements were abundant in *Eco*RI BESs. Repeat-sequence analysis revealed that the abundance of LINEs might be due to a GC bias of the restriction sites and that the GC content of silkworm LINEs was higher than that of mammalian LINEs. In a BLAST-based sequence analysis of the BESs against two available whole-genome shotgun sequence data sets, more than 70% of the BESs had a BLAST hit with an identity of ≥ 99%. About 14% of *Eco*RI BESs and about 8% of *Bam*HI BESs were paired-end clones with unique sequences at both ends. Cluster analysis of the BESs clarified the proportion of BESs containing protein-coding regions.

**Conclusion:**

As a result of this characterization, the identified BESs will be a valuable resource for genomic research on *Bombyx mori*, for example, as a base for construction of a BAC-based physical map. The use of multiple complementary BAC libraries constructed with different restriction enzymes also makes the BESs a more valuable genomic resource. The GenBank accession numbers of the obtained end sequences are DE283657–DE378560.

## Background

The silkworm (*Bombyx mori*) has been domesticated for more than 5000 years because of the industrial importance of sericulture. Besides being used for silk production, the silkworm is also an effective host for the production of recombinant proteins and biomaterials [1-3]. It is also an important model organism of the Lepidoptera, the insect order that includes the majority of serious agricultural pests. Therefore, the accumulation of silkworm genome resources will be helpful for both the control of agricultural pests and the development of the silkworm as an industrial-scale resource of biomaterials or bioreactors.

In silkworm, two individual whole-genome shotgun (WGS) projects have been carried out, and draft genomic sequences with 3× or 5.9× coverage have been generated [4,5]. Databases of expressed sequence tags (ESTs) and a single nucleotide polymorphism linkage map have also been released [6,7]. Bacterial artificial chromosomes (BACs) [8], as well as fosmids [9], also constitute important genomic resources. The main advantage of BACs, compared with yeast artificial chromosomes [10] or cosmids [11] is their higher stability, simplicity of construction and screening, low frequency of chimeric clones, and ease of DNA isolation. Therefore, BACs are one of the main tools used for high-throughput genomic studies, including for sequence-tagged connector (STC) strategies, BAC-based physical maps, and DNA fingerprinting, in various species [12-26].

BAC end sequences (BESs), single-pass sequence reads from each end of a BAC clone, are a powerful tool that enhances the value of BACs as a genomic resource [27-31]. We conducted large-scale BAC end-sequencing of two silkworm BAC libraries, the RPCI-96 *Bombyx mori *Silkworm P50 BAC Library [32] and the Texas A&M BAC Library [33], and characterized 94904 BESs.

## Results

### Sequence coverage

Two groups of BESs were obtained, one from the *Eco*RI-digested BAC library (*Eco*RI BESs) and the other from the *Bam*HI-digested BAC library (*Bam*HI BESs) (Table 1). The total length of the two BES groups was approximately 55 Mbp (Table 2). Given that the genome size of the silkworm is approximately 530 Mbp [34], the estimated sequence coverage of the *Eco*RI BESs and *Bam*HI BESs was 6.7% and 3.7%, respectively. Thus, by simple summation, the total sequence coverage was 10.4%.

**Table 1:** Summary of two bacterial artificial chromosome (BAC) libraries

	*Eco*RI-digested library	*Bam*HI-digested library
Vector	pBACe3.6 [52]	pBeloBAC11 [53]
Cloning site	*Eco*RI	*Bam*HI
Number of clones	36000 (96 × 384 wells)	21120 (55 × 384 wells)
Mean insert size (kbp)	168	165
Clone coverage	× 11.4	× 6.6
Strain	p50T (mixed insects)	p50T (mixed insects)

To calculate the percentage of the silkworm genome covered by the clones (clone coverage) in the *Eco*RI- and *Bam*HI-digested libraries, we assumed that the silkworm genome size was 530 Mb [34]. Detailed information on the *Eco*RI-digested library, such as the size distribution of BAC inserts, is available in the paper cited [49] and at the RPCI-96 BAC Library website [32]. Detailed information on the *Bam*HI-digested library can be obtained from the website of Texas A&M BAC Libraries [33].

**Table 2:** Characteristics of the two groups of BAC end sequences (BESs)

	*Eco*RI BESs	*Bam*HI BESs	Total
Number of sequences	61696	33208	94904
Average read length (bp)	571.6	598.1	580.9
Minimum read length (bp)	50	50	50
Maximum read length (bp)	955	920	955
Total bases (bp)	35266874	19860186	55127060
GC content (%)	37.45	40.30	38.47
Clones	34240	18251	52491
Paired-end clones	27456	14957	42413
Percentage of paired-end clones (%)	80.2	82.0	80.8

A paired-end clone is a clone that contains both end sequences. The percentage of paired-end clones is the ratio of the number of paired-end clones to the total number of clones.

### Repeat analysis of BESs

We estimated the transposable element (TE) content of the two sets of BESs. First, to construct a custom silkworm repeat database for use as a custom library file of the RepeatMasker program [35], we extracted silkworm repeat-related sequences enrolled in NCBI-GenBank (Release 152.0) [36] with a custom Perl script. All completely redundant sequences in the library except for a single representative sequence were then removed. The number of TEs in this library was 233. To mask repetitive sequences from each BES, we used RepeatMasker (version open-3-1-3) with default settings. Detailed information on the masked bases is provided in Table 3. The percentage of masked bases in the *Bam*HI BES group (21.3%) was higher than that in the *Eco*RI BES group (13.6%). Long interspersed nuclear elements (LINEs) predominantly accounted for this difference. To explain this difference between the two BES groups, we examined the bias of the two restriction enzymes. The average interval of recognition sites of *Eco*RI and *Bam*HI was 3.8 and 7.9 kbp, respectively, suggesting that in the silkworm genome *Eco*RI restriction sites were more abundant than *Bam*HI restriction sites. In addition, we estimated the GC% of the silkworm protein coding region to be 43.2%, based on silkworm protein coding sequences collected from GenBank, whereas the reported overall GC content of the silkworm genome is 32.54% [4]. Therefore, the GC% of *Bam*HI recognition sites (67%) is closer to that of the protein coding regions than to that of the genome as a whole. Conversely, the GC% of *Eco*RI recognition sites (33%) is closer to that of the genome as a whole. These results suggest the GC bias between the two restriction enzymes may explain the difference in the abundance of TEs between the two BES groups.

**Table 3:** Distribution of interspersed repeat DNA sequences within both BAC end sequences (BESs) in different repeat classes

	*Eco*RI BESs			*Bam*HI BESs		
	
	GC%	Elements	Percentage	GC%	Elements	Percentage
SINE	45.57	4088	1.63	45.92	2120	1.37
LINE	53.85	6865	5.14	53.22	11105	16.40
LTR	47.04	4140	2.32	47.26	2264	2.22
DNA	41.49	3711	3.77	40.77	744	0.70
Unclassified	40.91	1469	0.69	41.24	963	0.62

"Elements" denotes the number of repeat elements detected. "Percentage" denotes the ratio of length occupied by interspersed repeats to total length. GC content of unmasked region of *Eco*RI and *Bam*HI BESs were 35.49% and 37.05%, respectively. Overall GC content of *Eco*RI and *Bam*HI BESs were 37.45% and 40.30%, respectively.

To find novel repeat sequences in the BESs, we analyzed the repeat-masked BESs with RECON (version 1.05) [37], which automatically identifies *de novo *repeats. Only detected repeat families with 50 or more members were retained for further analysis. As a result, 31 and 15 repeat families with 50 or more members were detected in the *Eco*RI and *Bam*HI BESs, respectively. We then used BLASTX [38] to compare each repeat sequence against the nr (non-redundant protein) database, and found that 34.0% of the sequences had similarity to TE-related proteins. We used representative sequences of the repeat families for a BLAST search of silkworm whole-genome shotgun (WGS) data [4] to confirm whether they were really dispersed throughout the genome. The estimated copy number ranged from 9 to 2431; therefore, a large proportion of the detected sequences could be regarded as repetitive. However, a few sequences showed a much lower copy number than that estimated by RECON. It was recently reported that the great majority of silkworm transposon insertions are 5' -truncated, so most of the detected repeat sequences may be ''transposon fossils'' with no activity [4]. Further analysis of the detected sequences might reveal novel transposons in silkworm.

### BLAST search against whole-genome shotgun data

All BESs were used as queries in a BLAST similarity search of the two available sets of WGS data: the WGS data set deposited by the Silkworm Genomic Research Program [4] (abbreviated as "SGP data" in this paper) and the data set deposited by the Beijing Genomics Institute [5] (abbreviated as "BGI data"). In this search, the expectation value (-e option, a probability cutoff value) was set to 1e-5 and the -b option (number of database sequences to show) was set to 1000.

**Figure 1:**
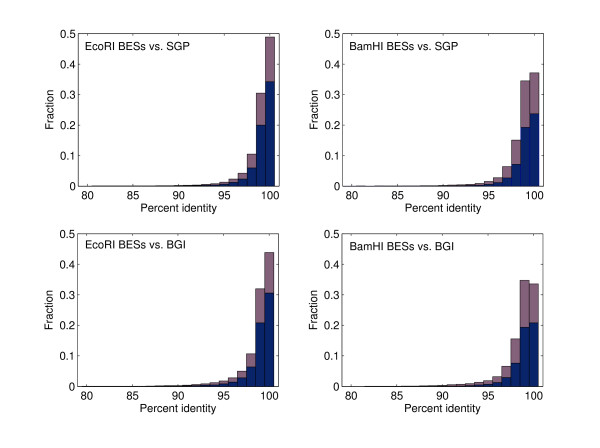
**Summary of BLAST searches with each group of BAC end sequences (BESs) versus the silkworm whole-genome shotgun sequencing (WGS) data sets**. BLAST searches were performed with each group of BESs against the two available silkworm WGS data sets. Each bin consists of two types of hits (red indicates a hit with, and blue a hit without, a repetitive region). The method for detecting a repetitive region was given in a previous section (Repeat analysis of BESs).

The percent identity distributions of BLAST hits (matched bases/aligned bases) between the BESs and the WGS data sets are summarized in Fig. 1. Although, the percent identity of the BLAST hits ranged from 80 to 100, the majority of BLAST hits (≥70%) showed ≥ 99% identity. Moreover, the BLAST hits of *Eco*RI BESs tended to have higher percent identity values than those of *Bam*HI BESs. This detected difference may reflect the higher abundance of repetitive sequences, which cause misassembly, in *Bam*HI BESs. The percent identity of BLAST hits against the SGP data also tended to be slightly higher than that against the BGI data. One possible cause of this difference may be strain divergence, because the BGI data were derived from an inbred domesticated silkworm variety, p50 (*Dazao*), whereas the SGP data were from strain p50T (*Daizo*), which diverged from p50 about 30 years ago and has been maintained at the University of Tokyo. To estimate the sequence divergence between the two data sets, the common and unique sequences were extracted from the two repeat-masked WGS data sets by BLAST-searching between them (e-value: 1e-50). BLAST hits containing bases within 200 bp of either end of WGS contigs were removed because the quality of sequences near the end of contigs can be relatively low. The percent sequence divergence calculated was too low to determine whether it was polymorphism-derived, considering that the estimated sequence error of the SGP and BGI WGS contigs is 0.08% and 0.045%, respectively [4,5]. Therefore, other factors such as sequencing errors in the WGS data sets might account for the difference in percent identity values between the two data sets.

**Figure 2:**
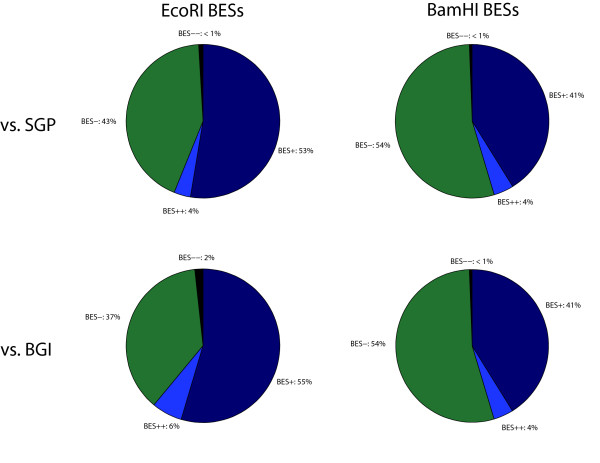
**BAC end sequence (BES) categorization results based on the BLAST search**. We defined a BLAST hit with ≥ 99% identity and > 0.8 alignment coverage, defined as the ratio of alignment length to BES length, as a match. BES+ denotes a BES with a single match, and BES++ a BES with multiple matches. BES- denotes a BES without a match, and BES-- a BES without a "raw BLAST hit."

We defined a match as a BLAST hit of ≥ 99% identity and ≥ 0.8 alignment coverage, which we defined as the ratio of alignment length to the BES length. The proportion of BESs with at least one match (that is, BES+ and BES++ sequences in Fig. 2) was greater with the BGI data than the SGP data. Conversely, BESs without matches (that is, BES- and BES-- sequences) were more abundant with the SGP data. The number of BES-- sequences common to both WGS data sets in the *Eco*RI and *Bam*HI BESs was 145 and 73, respectively. A BLAST search of BES-- sequences against ecoli.nt and vector databases revealed that 74 *Eco*RI and 34 *Bam*HI BES-- sequences were contaminated sequences, probably as a result of incomplete automated sequence trimming. The majority of the remaining BES-- sequences (69 *Eco*RI BESs, 31 *Bam*HI BESs) had no significant homology (e-value: 1e-05) in the nr or gss (genomic survey sequences) databases, indicating that they might be gap region sequences or sequences extraordinarily amplified during polymerase chain reaction (PCR) process.

The majority of BESs with a match were BES+, having only one match in each WGS data set. In addition, the percentage of ''multi-match'' *Eco*RI BESs (BES++ in Fig. 2) was lower than that of multi-match *Bam*HI BESs. We inferred each BES+ to be a unique region-derived sequence, and BES++ to be likely derived from repetitive sequences. We defined ''unique paired-end clones'' as paired-end clones showing a single match at each BES. A BLAST search of SGP data using the BESs as queries identified 8104 unique paired-end clones in the *Eco*RI library and 2778 among the *Bam*HI BESs. Similarly, a BLAST search of the BGI data yielded 8878 paired-end clones in the *Eco*RI BAC library and 3102 in the *Bam*HI BAC library. A total of 4757 unique paired-end clones among the *Eco*RI BESs, and 1482 among the *Bam*HI BESs, were common to both WGS data sets.

### BES clustering and coding region composition

We performed BES clustering, using "Combined BLAST and PhredPhrap" (CBP) as described in Methods, to examine BES composition in detail. Sequence clustering of each group of BESs was performed separately, using sequences of ≥ 100 bp. The percentage of singletons among the *Eco*RI BESs was higher than that among *Bam*HI BESs (Table 4).

**Table 4:** Summary of clustering results

Cluster size *d*	*Eco*RI BAC ends (%)	*Bam*HI BAC ends (%)
*d *= 1 (singleton)	28595 (71.69)	19731 (79.02)
4 > *d *≥ 2	9606 (24.08)	4306 (17.57)
8 > *d *≥ 4	1494 (3.75)	373 (1.52)
16 > *d *≥ 8	136 (0.34)	64 (0.26)
32 > *d *≥ 16	43 (0.11)	32 (0.13)
64 > *d *≥ 32	7 (0.0176)	9 (0.04)
128 > *d *≥ 64	5 (0.0125)	0 (0)
*d *≥ 128	1 (0.0025)	0 (0)
Total	39887	24515

Each representative sequence was then searched against the GenBank nr database (BLASTX, with the e-value set to 1e-05) to investigate the percentage of BESs containing protein-coding regions. As a result, 8068 clusters (20.2%) of *Eco*RI BESs had similarity to proteins in the database, compared with 6905 clusters (28.2%) of *Bam*HI BESs. For *Eco*RI BESs, most of the hit proteins were from *Bombyx mori *(53.8% of the clusters with similarity to proteins in the database), *Anopheles gambiae *(6.8%), *Apis mellifera *(4.0%), *Drosophila melanogaster *(3.2%), or *Bos taurus *(1.6%), whereas in the case of *Bam*HI BESs, most of the hit proteins were from *Bombyx mori *(68.4%), *Anopheles gambiae *(11.7%), *Apis mellifera *(8.5%), *Drosophila melanogaster *(2.4%), or *Bos taurus *(2.5%). The majority of large clusters showed similarity to TE-related proteins.

## Discussion

*Bam*HI BESs contained more repetitive sequences than *Eco*RI BESs. In particular, the two groups of BESs contrasted with regard to the abundance of LINEs. The GC bias of *Bam*HI may be main factor accounting for this difference because the GC% of *Bam*HI recognition sites was relatively close to the estimated GC% of protein coding DNA of the silkworm genome. This inference is further supported by the fact that the LINEs in the repeat sequences library had *Bam*HI recognition sites at average intervals of 2.0 kbp, whereas the average interval between *Eco*RI recognition sites was 3.0 kbp. These results indicate that the use of multiple BAC libraries constructed with different restriction enzymes can increase the genome representation [39].

The GC content of the masked region, especially the LINEs-derived region, was much higher than that of the unmasked region (Table 3). Conversely, the GC% of the DNA transposons-derived region was similar to that of the coding region. To confirm the GC-richness of silkworm LINEs, we calculated the GC content of each type of transposable element in the repeat sequences library and found that the median GC content of DNA-type elements (67 sequences), long terminal repeat (LTR) elements (30 sequences), LINEs (69 sequences), and short interspersed elements (SINEs) (26 sequences) was 39.1%, 43.7%, 51.9%, and 46.6%, respectively. Thus, the GC% of silkworm LINEs was rather higher than the estimated GC% of coding DNA of 43%. These results suggest that the GC richness of transposable elements, especially that of LINEs, primarily accounted for the greater abundance of TEs in the *Bam*HI BESs.

**Figure 3:**
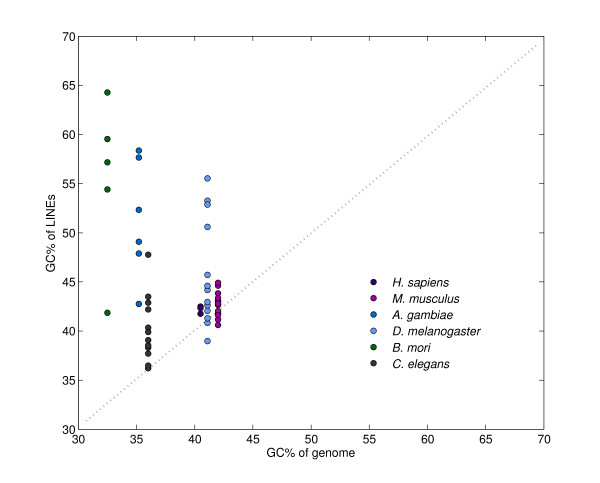
**Relationship between the GC% of genome and the GC% of long interspersed nuclear elements (LINEs) in different species**. We used the following LINE elements: *A. gambiae*; T1(M93689), RT1(M93690), RT2(M93691), Q(U03849), R6Ag3(AB090819), RTAg4(AB090813). *D. melanogaster*; BS(X77571), Doc(X17551), F(M17214), G(X06950), Helena(AF012030), HeT-A(U06920), I(M14954), Jockey(M22874), Pilger(AF278684), R1Dm(X51968), R2Dm(X15707), Tart(U02279), X(AF237761), You(AJ302712). *H. sapiens*; L1(U93574), HSLINE1O(X52235), L1.24(U93571), L1.21(U93570). *M. mulculus*; L1Md-A2(M13002), MMU15647(U15647), L1Md4(X14061), L1Md-Tf14(AF081108), L1Md-Tf23(AF081110), L1Md-Tf26(Af081112), L1Md-Tf9(AF081107), L1orl(D84391), L1spa(AF016099), L1Md-Tf18(AF0181111), L1Md-Tf30(AF081112), L1Md-Tf8(Af081106),, L1Md-Tf29(AF081113), L1Md-Tf17(AF081109), L1Md-Tf5(AF081104), L1Md-Tf6(AF081105). *B. mori*; BMC1(AB018558), R1Bm(M19755), R2Bm(AB076841), TRAS(AB04668), SART1(D85594). *C. elegans*; Rte-1(AF054983), Frodo-1(Z70755), Frodo-2(Z48009), Sam1(U13643), Sam2(U57054), Sam3(U46668), Sam4(Z92972), Sam5(Z81092), Sam6(Z82275), Sam7(Z82090), Sam8(AF016663), Sam9(Z81064).

Moreover, the GC richness of silkworm LINEs is notable because previous papers have reported that the AT-rich region of the mammalian genome contains an increased density of LINE insertions and mammalian LINEs have a relatively low GC content [40-44]. In general, LINEs of insects, especially silkworm, have a much higher GC content than those of mammals (Fig. 3). The GC richness of LINEs in silkworm might contribute toward the formation of specific genomic structures such as heterochromatin. A silkworm has a female heterogametic sex chromosome system (WZ/ZZ), as do most species of Lepidoptera. Moreover, the structural features of lepidopteran sex chromosomes have recently been described; that is, the W chromosome possesses a block of heterochromatin, which may comprise a small or a large segment of the chromosome or even the entire W chromosome [45]. The presence of many repetitive DNA elements in the W chromosome, especially non-LTR retrotransposons, has been reported [46,47]. These facts may suggest that silkworm LINEs are associated with the formation of heterochromatin. To further elucidate this possibility, analysis of more reliable genomic resources and cytogenetic methods is necessary.

The construction of a complete physical map is a vital task of genome sequencing projects. BESs are useful for identifying minimally overlapping clones that extend in each direction from finished clones. Unique paired-end clones are particularly useful for validating, ordering, and joining contigs. Therefore, BACs and their end sequences can be effectively used for integration of linkage and physical maps [12,28,29]. However, the possibility of mismapping, mainly due to sequence contamination must be considered. A BAC-based physical map can suffer from chimeric clones, genome assembly errors, and repetitive elements in the genome [48]. To reduce the incidence of incorrect mapping, tools such as repeat-masked BESs and BLAST searching with stringent criteria are necessary. In addition, DNA markers are helpful to detect incorrectly mapped clones. Contigs with two markers from different linkage groups should be tested for clone contamination [25]. Incorrect mapping can also be detectable as an inconsistency in the physical map when a deep coverage BAC library is used. This BLAST-based analysis revealed that the majority of BESs had BLAST hits with ≥ 99% identity against two available WGS data sets. Moreover, the percent identity of BLAST hits against BGI data tended to be slightly lower than that against SGP data, although the main cause of this tendency could not be determined by our analysis. The estimated sequence divergence between the p50T and p50 strains was too low to determine whether the divergence was polymorphism-derived. Therefore, merging of the two WGS data sets is reasonable and will contribute to the construction of a more useful genomic resource in the future.

## Conclusion

Characterization of BESs from two BAC libraries confirmed that BAC libraries by nature tend to have certain biases. Therefore, BESs from multiple complementary BAC libraries constructed with different restriction enzymes are a more useful genomic resource. The BESs produced by this research constitute a valuable resource for genomic research in *Bombyx mori*, for example, as a base for construction of a BAC-based physical map and for exploration of DNA makers. The GenBank accession numbers of the obtained end sequences are DE283657–DE378560.

## Methods

### Silkworm strain

We used the inbred silkworm strain p50T for the research.

### BAC libraries

We used two silkworm BAC libraries for end-sequencing, One library was constructed from a partial *Eco*RI (EC 3.1.21.4) digest of genomic DNA. The construction of this library was reported previously [49]. Copies are available through BACPAC RESOURCES at the Children's Hospital Oakland Research Institute [32]. The other library, prepared by using *Bam*HI as the restriction enzyme (EC 3.1.21.4), was purchased from the Laboratory for Plant Genomics and GENEfinder Genomic Resource of Texas A&M University [33]. The properties of the two BAC libraries are summarized in Table 1.

### Purification of BAC clones

*Escherichia coli *cells harboring single BAC clones were maintained at -80°C. A fresh colony from each clone was inoculated into each well of a 96-deep-well plate filled with 1.25 mL of 2× LB medium (2% tryptone peptone, 1% yeast extract, and 1% sodium chloride) containing 20 μg/ml chloramphenicol. They were cultured with shaking for 18 to 20 h at 37°C. BAC DNA was prepared using an automated DNA isolation system (PI-1100, Kurabo Industries Ltd., Osaka, Japan) according to the manufacturer's instructions.

### Sequencing of BAC ends

Sequencing reactions were performed with 3 μL Big Dye terminator mix (Applied Biosystems, Foster City, CA, USA), 1.0 μL 5× sequencing buffer, 0.5 to 1.0 μg template DNA, 10 pmol of primer, and 4 mM MgCl_2_. The conditions for the thermal cycling reactions were 96°C for 5 min, then 99 cycles of 96°C for 30 s, 55°C for 10 s and 60°C for 4 min, followed by holding at 4°C. We used custom T7 and SP6 sequencing primers. The DNA was recovered by using MultiScreen 384SEQ plates (Millipore, Billerica, MA, USA).

### Sequence trimming

Base-calling and trimming of BESs were performed with RAMEN, which was used for vector-trimming of silkworm WGS sequences [4]. A BLAST search of mtDNA sequences among the BESs was performed to identify and discard contaminated sequences (e-value: 1e-50). The obtained BESs have been deposited in the DNA Data Bank of Japan/European Molecular Biology Laboratory/GenBank under accession numbers DE283657 to DE378560.

### BES clustering

BES clustering was done with the in-house program "Combined BLAST and PhredPhrap" (CBP), which was developed mainly for clustering silkworm ESTs. This program internally uses BLAST [38] and PHRAP [50,51]. To optimize the clustering of the BESs, we modified the algorithm slightly. An outline of the clustering procedure follows.

**Step 1 **An all-to-all BLAST (BLASTN) operation of the BESs was performed. The expectation value (-e option) was set to 10, and no complexity filter (-F option) was used. The number of alignments to be reported (-b option) and maximum number of sequence bases to be created in a volume (-v option) were set to 1000000.

**Step 2 **Each BLAST hit was analyzed. A provisional cluster was created when a BLAST hit had an identity of at least 90% (*T*_pid_) and an alignment length of 90 bp (*T*_aln_). The longest sequence in each provisional cluster was chosen as the representative sequence. A provisional cluster of size 1 was treated as a "singleton."

**Step 3 **Sequences in each provisional cluster were assembled with PHRAP (using default parameters).

**Step4 **Reclustering and reassembling were performed under more stringent conditions if multiple contigs were generated. This process was iterated until a single contig was generated. For each iteration, the criterion of alignment length *T*_aln _was incremented by 30 bp if *T*_aln _was less than or equal to 300 bp. If *T*_aln _was greater than 300 bp, the incrementation of *T*_aln _was set to 15 bp. If a single contig was not generated by these iterations, then this process was iterated with a stricter *T*_pid _criterion until a single contig was generated. Any unassigned sequences were collected and stored for Step 6.

**Step 5 **Each contig generated in Step 4 was searched against the member sequences of its own contig for verification. Contigs that did not satisfy the condition, identity ≥ 95% and coverage of alignment ≥ 90%, were stored for Step 6.

**Step 6 **All sequences stored during the above steps were reprocessed (return to Step 2).

## Authors' contributions

YS designed the study, carried out the primary analysis, and wrote the majority of the text. HM and MS participated in developing the clustering software and the in silico analysis. SS and JN conducted the laboratory experiments, such as BAC end-sequencing. KM and KY supervised the research, participated in the design of the study and the interpretation of the data, and helped to draft the manuscript. All authors have read and approved the final manuscript.
